# Predictors of compliance with COVID-19 related non-pharmaceutical interventions among university students in the United States

**DOI:** 10.1371/journal.pone.0252185

**Published:** 2021-06-18

**Authors:** Spencer G. Shumway, Jonas D. Hopper, Ethan R. Tolman, Daniel G. Ferguson, Gabriella Hubble, David Patterson, Jamie L. Jensen

**Affiliations:** 1 Department of Chemistry and Biochemistry, Brigham Young University, Provo, Utah, United States of America; 2 Department of Biology, Brigham Young University, Provo, Utah, United States of America; 3 Department of Plant and Wildlife Science, Brigham Young University, Provo, Utah, United States of America; Bucharest University of Economic Studies, ROMANIA

## Abstract

The world is currently dealing with a devastating pandemic. Although growing COVID-19 case numbers, deaths, and hospitalizations are concerning, this spread is particularly alarming in the United States where polarizing opinions, changing policies, and misinformation abound. In particular, American college campuses have been a venue of rampant transmission, with concerning spillover into surrounding, more vulnerable, communities. We surveyed over 600 college students from across the United States and modeled predictors of compliance with non-pharmaceutical interventions. We identified concern with severity, constitutionalism, news exposure, and religiosity as significant positive correlates with compliance, and general trust in science as a significant negative correlate. To determine how applicable nationwide modeling might be to individual local campuses we also administered this same survey to nearly 600 students at two large universities in Utah County. In this population, concern with severity was the only significant positive correlate with compliance; Additionally, feelings of inconvenience were negatively correlated. The effects of feelings of inconvenience, and news exposure were significantly different between populations. These results suggest that we should focus our efforts on increasing knowledge about the pandemic’s effects on our society and informing about constitutionality amongst college students. However, we also show that nationwide surveys and modeling are informative, but if campuses are to efficiently curb the spread of COVID-19 this coming semester, they would be best served to utilize data collected from their student populations as these might significantly differ from general consensus data.

## Introduction

### Background on pandemic

The emergence of the novel coronavirus Sars-Cov-2, which causes the disease COVID-19, rapidly spread from an outbreak to a global pandemic [1]. When these data were collected (October and November 2020), the World Health Organization had reported nearly 56 million confirmed cases, and over 1.3 million deaths globally [2], and other estimates had reported much higher numbers in both categories [3]. Additionally, the subsequent economic [4] and psychological [5] fallout from the pandemic had been felt across the globe.

Experts have suggested that increased compliance with non-pharmaceutical interventions could have reduced the consequences of the pandemic [6, 7], and increased compliance in the future could save tens of thousands of lives in America alone [3, 8, 9]. Non-pharmaceutical interventions include wearing masks, practicing social distance, washing hands, and sanitizing surfaces, among other things. A CDC study from 2008 found that most Americans would be willing to practice mitigation measures to slow the spread of a pandemic influenza [10], but this is not the reality that is seen today. Despite the scientific evidence behind the efficacy of interventions such as masks [6, 8, 11–13], social distancing [11] and increased handwashing [11], many Americans refuse to take these precautions [6, 14–20].

The spread of COVID-19 has been of particular concern on university campuses. Again, when these data were collected, there had been over 321,000 cases at over 1,700 American colleges and universities [21]. A study looking at Fall 2020 classes has shown that counties with large universities with in-person instruction had a large increase in COVID-19 cases in the three weeks after school started, while counties with universities with remote instruction had a decrease in number of cases during this similar time frame [22]. While college students are typically at a low risk for severe infection and death, spread of COVID-19 on campuses has the potential to spill over into more vulnerable communities adjacent to campuses [23, 24]. Additionally, long-term consequences of COVID-19 infections are not well understood [25], thus widespread infections amongst college students could plausibly lead to complications in the future.

As of November 19, 2020, the state of Utah had reported nearly 166,000 total COVID-19 cases, 7,215 hospitalizations, and 756 deaths [26]. Doctors and public health officials warned of the strain on Utah’s healthcare system, as ICU capacity has been exceeded in several major hospitals across the state [27–30]. Two weeks after seeing a spike in cases, Utah’s governor declared a “COVID-19 State of Emergency” [31]. Overall, 8,881 cases had been reported at 10 universities and colleges, however 2,993 had been reported at one of the state’s larger universities [21]. During the time the survey in this study was administered, the United States had approximately 9.3 million total cases of COVID-19, and Utah County had reported approximately 30,000 cases [26].

Work has been conducted in the United States and internationally to identify predictors of compliance with non-pharmaceutical interventions. On an international level Clark et al. [32] found that a belief in the effectiveness of precautionary measures was a significant predictor of whether individuals took precautions. Plohl and Musil [33] found that COVID-19 risk perception and trust in science were able to independently predict compliance, and political conservatism, religious orthodoxy, conspiracy ideation and intellectual curiosity were found to be predictors via the mediating role of trust in science. In America, political conservatism and party identification have consistently been identified as predictors of precautionary behaviors; those who identify as conservative and/or Republican are the least likely to be compliant, while those who identify as liberal and/or Democrat are most likely to be compliant [15–20, 34].

While there has been some modeling of compliance with non-pharmaceutical interventions (referred to as NPIs for the remainder of this paper) amongst the general population, to our knowledge there has not been any modeling of compliance amongst undergraduate students, one of the populations that is contributing to the spread of the current pandemic. In this study we use structural equation modeling to identify the predictors of compliance amongst a subset of university students from across the country, and at large campuses in Utah County that, as of the time of submission, were experiencing a large amount of community spread among its students.

## Materials and methods

### Ethics statement

Permission for this study was obtained from the institutional review board at Brigham Young University. Participants gave their implied consent by filling out our survey. No minors were involved in this study.

### Study population

The nationwide survey was administered through the Qualtrics survey platform, with parameters given to match the national profile of undergraduate students as reported by the national center for education statistics [35]. Qualtrics surveys broadly nationwide but allows for filters to be placed on respondents so that responses are recruited and eliminated until quotas are met. Our filters were as follows: We targeted students currently enrolled in a college or university, including public, private, and trade schools; participant age had to be 18 or above; we recruited approximately 50% male and 50% female, although ‘other’ was also included in analysis; race/ethnicity was selected based on national averages of approximately 66% non-hispanic white, 12% non-hispanic black, 12% hispanic, and 10% other; and we recruited approximately 50% of the sample on the left-side of the politically ideology spectrum, and 50% on the right side. Given these filters and a request for approximately 600 responses from each population [based on the general rule of thumb of between 5 and 10 responses per parameter measured, and an approximate 70-item measurement model [36, 37] and taking into account our demographic parameters), we obtained 608 responses in our national sample. The local survey (Utah Valley) was administered to students enrolled in both general non-majors biology classes and entry level majors biology classes at two Universities in Utah, one private institution with enrollment around 35,000 students and one public institution with open-enrollment around 41,000 students, both located in Utah County. All students in these classes were recruited by incorporating our survey into a homework assignment worth a small number of points. Students who opted not to participate were given the opportunity to complete an alternate assignment for the equivalent number of points. Again, targeting approximately 600 responses, but constrained by class sizes, we obtained 566 responses in our local sample. We then excluded individuals over the age of 39 (i.e., we included millennials and younger) in order to eliminate outliers who were not necessarily representative of the typical college student. This left us with 547 in the General population and 595 in the Utah County population.

### Survey development

#### Literature review and interviews

To begin construction of our instrument we surveyed literature about predictors of COVID-19 compliance, and trust in science in general. We found 5 factors shown to be predictors of compliance (Table 1), and 3 plausible factors based on their influence on trust in science generally (Table 2).

**Table 1:** Factors shown to influence compliance with non-pharmaceutical interventions.

*Predictor*	*References*
*Political Ideology*	[15–20, 33]
*Trust in Science and Scientists*	[19, 33]
*Geographic Location*	[14, 34]
*Trust in Gov’t Handling of Pandemic*	[38]
*Fear of racial profiling*	[39–43]

**Table 2:** Plausible predictors of compliance.

*Predictor*	*References*
*Vaccine hesitancy*	[44–46]
*Acceptance of Evolution*	[47–49]
*Religiosity*	[47, 49, 50]

We then conducted qualitative interviews with undergraduate students at one of the Utah County schools to refine our instrument and test for validity. We asked students questions about their age, major, political ideology, party identification, news source and exposure to news, feelings regarding the government’s response to the pandemic, practice of NPIs, trust in science generally, trust in medical science, trust in public health officials and their doctor, religiosity, and acceptance of organic evolution. Interviews were transcribed using software from temi.com and responses were coded by three students independently, who then collaboratively agreed upon a score for each part of the interview transcript. Our interview script was based heavily on published instruments modified to interview form (e.g. [51, 52]). While we recognize we interviewed too few students for meaningful data to be obtained, we feel clear themes were noticed in the interview data. A few things we asked in the interviews we deemed unnecessary as responses were uninteresting and unvaried. For example, we asked about handwashing procedures, and responses were vague and similar among participants. We asked about trust in public health officials and trust in professional medical professionals separately, then due to similarity in responses we combined these questions for our survey. We did a similar thing for the religiosity section of the survey. In the interview we asked about trust in science briefly and we expected more explanatory responses from the participants than we got. Partially due to this response we added a longer trust in science instrument for our survey [51]. We asked those we interviewed how they felt local and national governments were doing handling the pandemic. These responses were relatively uninformative and questions for the survey were changed to ask about feelings of constitutionality. Although a lot was changed between the interview script and the published survey, the majority of changes were formatting changes to fit the criteria of a survey.

We developed two surveys, one to be administered to students in Utah Valley, and another that was sent to students nationwide. They were largely identical, with the exception being that the nationwide survey asked students what type of institution they attended and their gender, while the Utah Valley instrument asked respondents which university they attended. We settled on the following demographics and latent variables.

### Demographic measures

We measured participant age, the type of institution the student attends (i.e., public/private, 4-year institution, 2-year institution, trade school), where the students grew up, where the students are currently attending college, and what year of college they are in. In addition, we asked for student identified gender (only in the nationwide survey) as well as political party identification (Democrat, Republican, Independent, or Other).

### Latent variables

We identified and measured the following latent variables:

#### Political ideology

To measure political ideology students were asked to rate their view of economic, social and scientific views on a scale of 1 (meaning very left leaning or liberal) to 7 (meaning very right leaning or conservative).

#### Religiosity

To measure participant religiosity we chose to use the Duke Religiosity Index (DUREL) [52] due to its brevity, and optimization for use in public health surveys. Question one was changed from, *How often did you attend church or other religious meetings*? to, *Before the emergence of COVID-19*, *how often did you attend church or other religious meetings*? We felt that attendance at church or other religious meetings could currently be influenced by local mandates about gatherings and fear of contracting COVID-19, and may not be an indicator of religiosity at the present time. All other questions were coded and worded as reported by Koenig et al. [52].

#### Religiosity and COVID

Participants were asked to answer which response most accurately described their view of each of the following statements with a scale from 1 to 5 (*Definitely not true*, *tends not to be true*, *unsure*, *tends to be true*, *definitely true of me*): *My religion influences my beliefs about the current COVID-19 pandemic; My religion influences my approach to the current COVID-19 pandemic;* and *My religious beliefs shape how I have interacted with others during the COVID-19 pandemic*. This scale was created by the authors, and no items were reverse coded.

#### General trust in science

For this section, we consulted Nadelson et al. [51]. Their twenty-one-item instrument is designed to assess the level of trust an individual has in the scientific method and scientists. The concept of trust is difficult to define, and even more difficult to measure. However, based on the literature, we believe trust in science to be one of the best predictors of compliance with recommended COVID-19 health practices. Furthermore, we believe that the Nadelson et al. instrument adequately assesses a person’s trust in science.

Participants were asked to answer the following questions on a 5-pt Likert-type scale:

1. Scientists ignore evidence that contradicts their work.
2. Scientific theories are weak explanations.
3. Scientists intentionally keep their work secret.
4. Scientists don’t value the ideas of others.
5. We should trust the work of scientists.
6. We should trust that scientists are being honest in their work.
7. We should trust that scientists are being ethical in their work.
8. Scientific theories are trustworthy.
9. We can trust science to find the answers that explain the natural world.
10. We cannot trust scientists because they are biased in their perspectives.
11. Scientists will protect each other even when they are wrong.
12. We cannot trust scientists to consider ideas that contradict their own.
13. Today’s scientists will sacrifice the well being of others to advance their research.
14. We cannot trust science because it moves too slowly.

We removed questions 1, 5, 7, 8, 13, 14, and 16 from the original Nadelson instrument based on rationale provided by Plohl and Musil [33].

#### Trust in public health officials

The trust in public health officials is a modification of Nadelson et al. [51]. We rephrased the questions to use the phrase ‘public health authority’, which we defined as follows: “For this next section, you will see the term ‘public health authority’ used a number of times. We are using this term to mean individuals employed by tax-funded government health agencies, whether that be local, state, or federal. Specifically, a public health authority communicates with the public with statements about health concerns and recommended health practices. A prominent example would be Anthony Fauci, M.D., director of NIAID (National Institute of Allergy and Infectious Diseases).” We believe this definition to provide a clear and easily understood definition of the term, as well as providing a colloquial example for reference. We believe ‘public health authority’ is a politically weighted term that is commonly used. Participants were asked to answer the following questions on a 5-pt Likert-type scale:

1. When public health authorities provide conflicting advice, it diminishes my trust in their work.
2. Public health authorities ignore evidence that contradicts their work.
3. Public health authorities intentionally keep some information secret.
4. Public health authorities are seeking to fulfill an agenda.
5. Public health authorities don’t value the ideas of others.
6. I trust that public health authorities want to make life better for people.
7. We should trust that public health authorities are being honest in their work.
8. We should trust that public health authorities are being ethical in their work.
9. When public health authorities give health instruction, they are just guessing.
10. We cannot trust public health authorities because they are biased in their perspectives.
11. We cannot trust public health authorities to consider ideas that contradict their own.
12. Today’s public health authorities will sacrifice the well-being of others to advance their agenda.

In question 12, the word “research” was changed to “agenda” from the original Nadelson et al. instrument. We removed questions from the original Nadelson instrument that did not fit with our topic of focus in the interest of clarity and brevity, leaving us with 12 questions.

#### Priorities of public health officials

Blendon et al. [10] asked respondents which of a public health official’s duties was most important. For comparison to understand how attitudes towards public health officials may have shifted, we repeated this question, and instead asked respondents to rank the following priorities of public health officials: to *Treat everyone as equally as possible; Protect the health of the greatest number of people; Give priority to sick and frail people in getting attendance; Aim to preserve essential community services like electricity and law enforcement;* and *To not interfere with the civil liberties or freedoms of people in your communities*.

#### Constitutionalism

Participants were asked to answer which response was closest to their view of each of the following statements on a five-point Likert scale (*Definitely not true*, *tends not to be true*, *unsure*, *tends to be true*, *definitely true of me*): *I don’t mind masks*, *but mask mandates are unconstitutional; Mask mandates interfere with my constitutional rights; Mask mandates interfere with my personal freedoms; COVID-19 restrictions restrict my constitutional rights; I have the constitutional right to refuse to wear a mask in public;* and *Wearing a mask should be a personal choice*, *not a legal mandate*. We created this section of the questionnaire because we found in our preliminary interviews that constitutionalism was a common theme for those who refused to wear a mask, and we hypothesized that many only refused to wear a mask because of their ideas about their constitutional rights. It’s worth noting that this variable primarily identifies issues subjects had with mask-wearing. Other NPIs were not included in the questions.

#### Convenience

Many of our questions for the convenience portion of the survey were based on the information that was found in a survey sent out by YouGov [15]. This survey was intended to examine attitudes around face masks among voters in the United States. The questions were slightly modified to fit our needs. Participants were asked to rank the following statements on a 5-point Likert scale (*Definitely not true*, *tends not to be true*, *unsure*, *tends to be true*, *definitely true of me*): *The discomfort of a mask is enough to make me not wear them; Physical issues (e*.*g*., *glasses fogging up*, *facial breakouts*, *difficulty breathing) prevent me from wearing a mask; The lack of availability of masks prevents me from wearing one;* and *Forgetting to bring a mask with me is often a reason I don’t wear one*.

#### Concern with severity

To test for perceived impression of severity of the COVID-19 virus we asked four questions modified from a 2007 study on barriers to flu vaccines [53]. In that survey respondents were asked: “How concerned are you about getting the flu?” and “How would getting the flu affect your life?”. Both of these questions had three possible answer choices. We changed the answer scale to a five-point scale to be consistent with the rest of the survey. We changed the wording of their questions to apply to COVID-19, for example, instead of asking, “How concerned are you about getting the flu?”, we asked “How concerned are you with getting COVID-19?”. We separated the second question into two questions and adjusted it to be COVID-19 specific. We asked about how they felt the novel coronavirus could affect them physically, and how they thought it could affect other aspects of their life. We added one more question asking about how they felt the novel coronavirus would affect others in their community. This question was added to give us the greater statistical power associated with four questions instead of two.

#### Personal responsibility

In a thorough search of the literature no studies were found that correlated personal responsibility to a role in compliance with non-pharmaceutical methods of decreasing viral infection. We did find one study that researched public risk perceptions, personal responsibility, and self-isolation habits during the recent COVID-19 pandemic [54]. As they mostly focused on self-isolation habits, no survey instrument from their study could reasonably be used in our survey. Another study analyzes how different social norms including both impression of severity and personal responsibility affect compliance to precautionary guidelines, but this study didn’t involve surveys of any kind, thus, no instrument was developed [55]. We mimicked the way questions were asked in the Baal research study and asked our respondents to rank the following five statements on a seven-point Likert scale (from Strongly Disagree to Strongly Agree): I shouldn’t be mandated to wear a mask because those who are at high risk for contracting the virus should be responsible for keeping themselves safe; I am responsible for the health of others in my community*; Everyone should take responsibility for their own health and act accordingly; Those who are at high risk should stay out of public places, *rather than having everyone else take precautions for their health;* and I should feel responsible and take action to protect the health of those most vulnerable in the population* (with asterisks indicating reverse-coded items).

#### Exposure to news

We formulated our own questions regarding news exposure based on the results from our preliminary interviews. In the interviews, we asked the questions, “Do you keep up with science-related news?” and, “Do you keep up with COVID-19 related news?” Students had various responses to how well they kept up with these specific news types, so we decided to gauge how often they checked both science and COVID-19 related news on a 6-point Likert scale of everyday to never. Additionally, to gauge how often students kept up with non-science and COVID-19 related news, we used the same scale (everyday to never).

#### Precautionary behaviors

After conducting the preliminary interviews, we found that students had given various responses on their extent of mask wearing, social distancing, and hand washing. For mask wearing, we found that there were certain situations where students were more or less likely to wear masks. From this, we asked students to rank their mask wearing, on a scale of always to never, for three distinct situations: where masks are required, in small groups of individuals not of their immediate household, and in large groups and outdoors within six feet of others. Additionally, we created questions using the same scale (always to never) to address the extent of social distancing, hand washing, and sanitizing nearby surfaces (the full survey is available in the S1 File).

### Statistical analysis

Each population (General and Utah County) was analyzed separately. We conducted confirmatory factor analysis (CFA) on each of measurement model for each latent variable (or on combined models with latent variables containing less than four items). We used modification indices to allow errors to covary between items and removed items with factor loadings less than 0.40 and *p* < .05 identifying them as weak indicators of our factors.

We used several fit indices, including Tucker-Lewis index (TLI), comparative fit index (CFI), and root mean square error of approximation (RMSEA). We then tested for measurement invariance (metric and scalar) between populations. Because neither metric or scalar invariance was met, Structural Equation Modeling (SEM) was performed on each individual full dataset (General and Utah County) to create two models [56]. CFA and SEM procedures were performed using the Mplus software, version 8, utilizing the Robust Maximum Likelihood (MLR) estimator.

### Comparison between populations

Comparisons in age and year in school between populations was done using Wilcoxon Signed-Rank Tests.

## Results

### Interview results

While our interview sample size was small, we observed the following patterns which are reflected in our instrument:

- Those who specifically mentioned that their religiosity influenced their attitude about the pandemic tended to take NPIs more seriously, even when compared to those who had a high religiosity in general.
- View of governmental response was captured by political party and ideology.

### Confirmatory factor analysis

**Fig 1:**
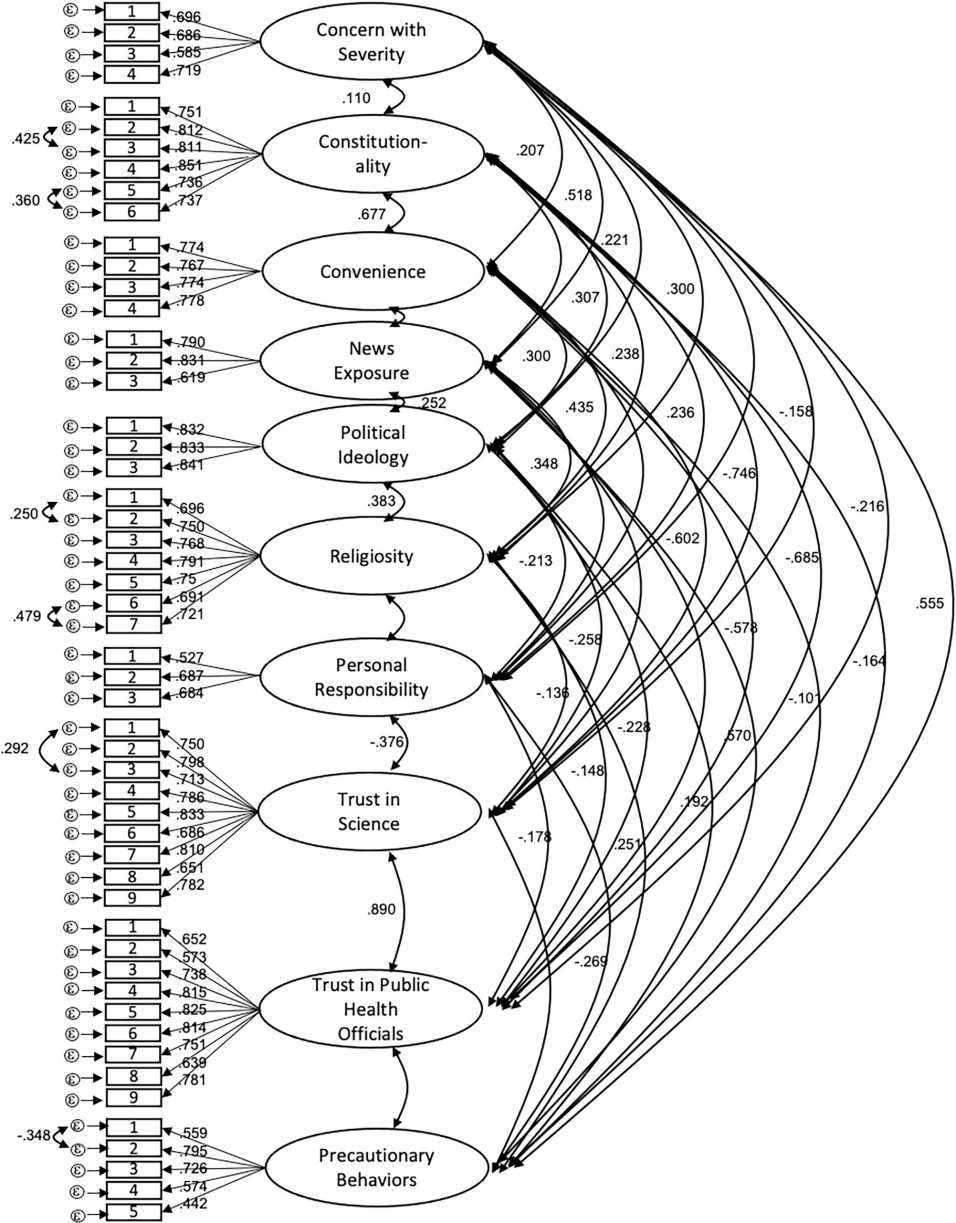
Culminating measures model for the general population. Culminating measurement model for the General population. Bidirectional arrows indicate correlation coefficients; directional arrows indicate standardized factor loading values for each item on their respective factor. All correlations listed were significant at *p* < .05. All standardized factor loadings were significant at the *p* < .01 level.

**Fig 2:**
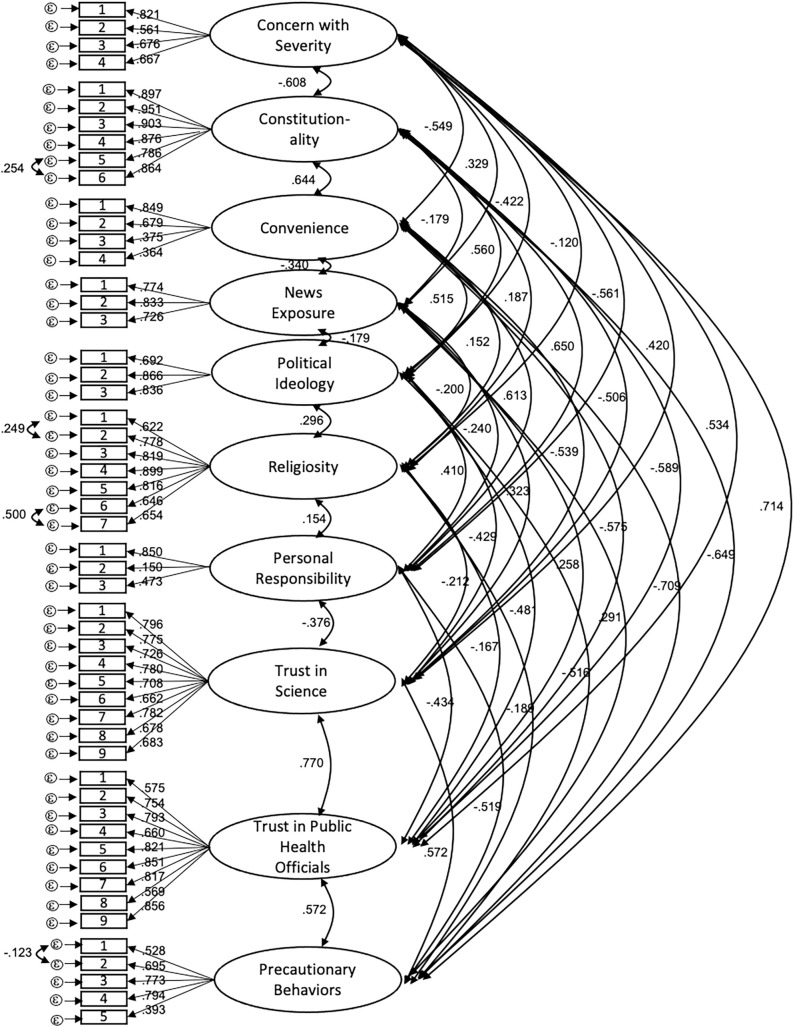
Culminating measures model for the Utah population. Bidirectional arrows indicate correlation coefficients; directional arrows indicate standardized factor loading values for each item on their respective factor. All correlations listed were significant at *p* < .05. All standardized factor loadings were significant at the *p* < .01 level.

CFAs within each population showed acceptable fit of the data to the proposed model. High correlation between items indicated that the General Religiosity and Religiosity and COVID instruments should be combined into one latent variable that we labeled Religiosity. Fit statistics are shown for each population in Table 3. Our full measurement models for each population are shown in Figs 1 and 2. Tests for measurement invariance failed to show metric or scalar invariance between populations (fits statistics are shown in Table 3) as CFI values increased by more than 0.01 and AIC measures increased between models. Correlations between factors in the model in each population are shown in Tables 4 and 5.

**Table 3:** Fit statistics for CFA in each population and models for measurement invariance.

Population	RMSEA	CFI	TLI	SRMR	AIC
General	0.036	0.936	0.931	0.053	83636.47
Utah	0.031	0.956	0.952	0.044	76312.61
Configural	0.033	0.946	0.942	0.048	159949.08
Metric	0.037	0.931	0.926	0.062	160414.64
Scalar	0.044	0.901	0.897	0.072	161336.59

**Table 4:** Correlation matrix of latent variables for the general model.

*Measure*	1	2	3	4	5	6	7	8	9
1. Concern with severity	_								
2. Constitutionality	.110^a^	_							
3. Convenience	.208	.677	_						
4. Personal Responsibility	-.121^b^	.236	.106^b^	_					
5. News Exposure	.518	.057^b^	.037^b^	-.112^b^	_				
6. Political Ideology	.221	.307	.300	-.213	.525	_			
7. Religiosity	.300	.238	.435	-.073^b^	.348	.384	_		
8. General Trust in PHOs	-.216	-.685	-.578	-.178^a^	-.035^b^	-.227	-.148^a^	_	
9. General Trust in Science	-.158^a^	-.746	-.602	-.201^a^	.006^b^	-.258	-.136^a^	.890	_
10Precautionary Behaviors	.555	-.164	-.100^a^	-.269	.571	.192	.251	.054^b^	.058^b^

Note: All correlations are significant at *p* ≤ .001 unless specified

^a^ indicates *p* < .05

^b^ indicates non-significance.

**Table 5:** Correlation matrix of latent variables for the Utah County model.

*Measure*	1	2	3	4	5	6	7	8	9
1. Concern with severity	_								
2. Constitutionality	-.606	_							
3. Convenience	-.546	.642	_						
4. Personal Responsibility	-.562	.649	.610	_					
5. News Exposure	.341	-.190	-.346	-.247	_				
6. Political Ideology	.514	.657	.618	.482	-.217	_			
7. Religiosity	-.119^a^	.187	.152	.154^a^	-.197	.306	_		
8. General Trust in PHOs	.359	-.424	-.423	-.327	.271	-.636	-.213	_	
9. General Trust in Science	.299	-.383	-.359	-.282	.131^a^	-.584	-.213	.372	_
10. Precautionary Behaviors	.706	-.640	-.702	-.516	.308	-.608	-.196	.474	.335

Note: All correlations are significant at *p* ≤ .001 unless specified

^a^ indicates *p* < .05.

### Initial SEM model

**Fig 3:**
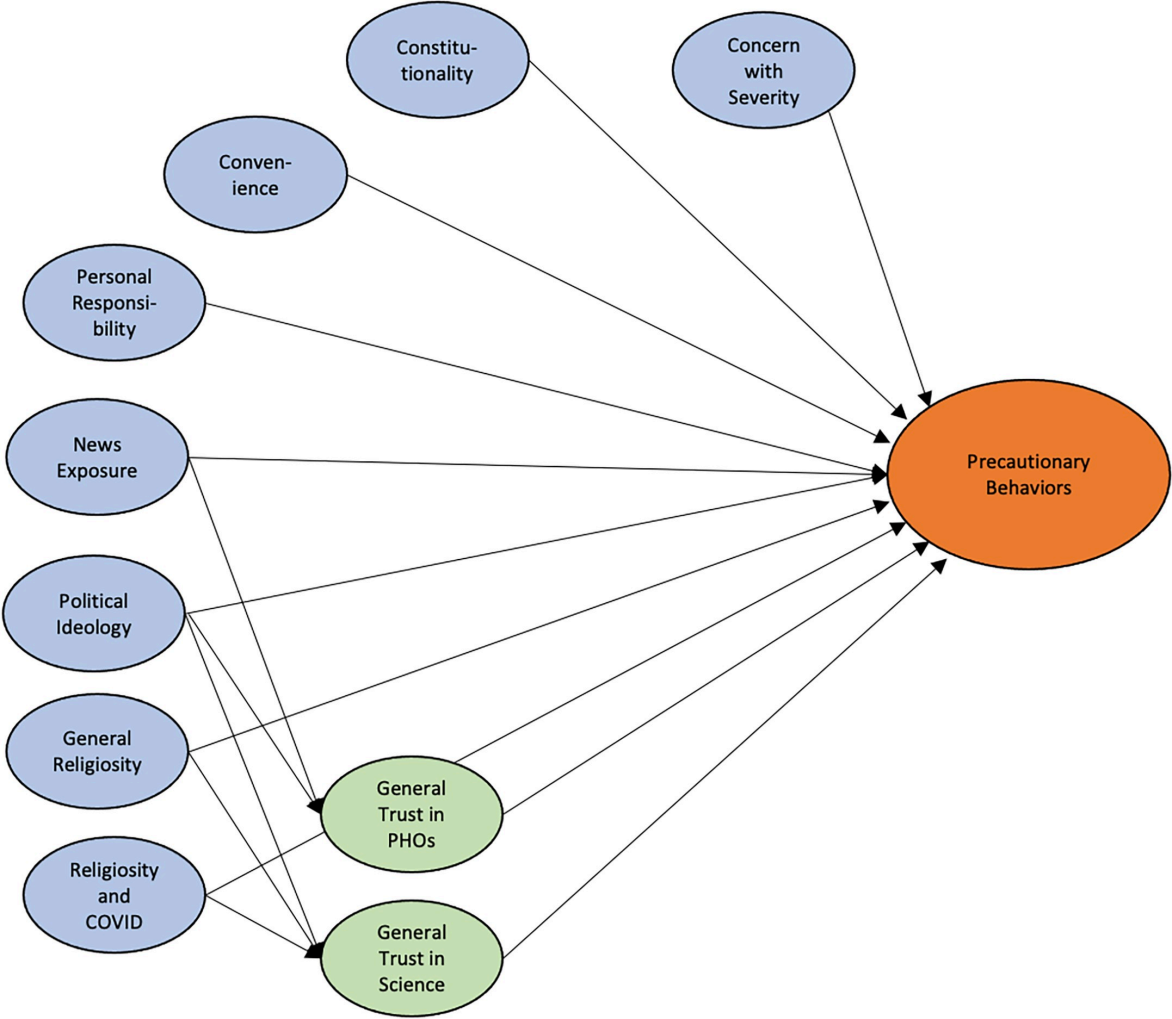
Hypothesized SEM model. The hypothesized structural equation model that characterizes relationships between factors. Lines indicate predictive relationships.

Based on our interviews and literature surveys we developed the statistical model shown in Fig 3. We hypothesized that impressions of severity, belief in the importance of personal responsibility, belief in personal liberty, feelings of inconvenience, news exposure, political ideology, religiosity, religious views on the pandemic, trust in science and trust in public health officials would all directly influence taking precautionary behaviors. We also hypothesized that political ideology, religiosity, and the influence of religious views on the pandemic would influence precautionary behaviors through the intermediate of trust in science; and news exposure and political ideology would affect precautionary behaviors through the mediating factor of trust in public health officials.

### Structural equation modeling

**Fig 4:**
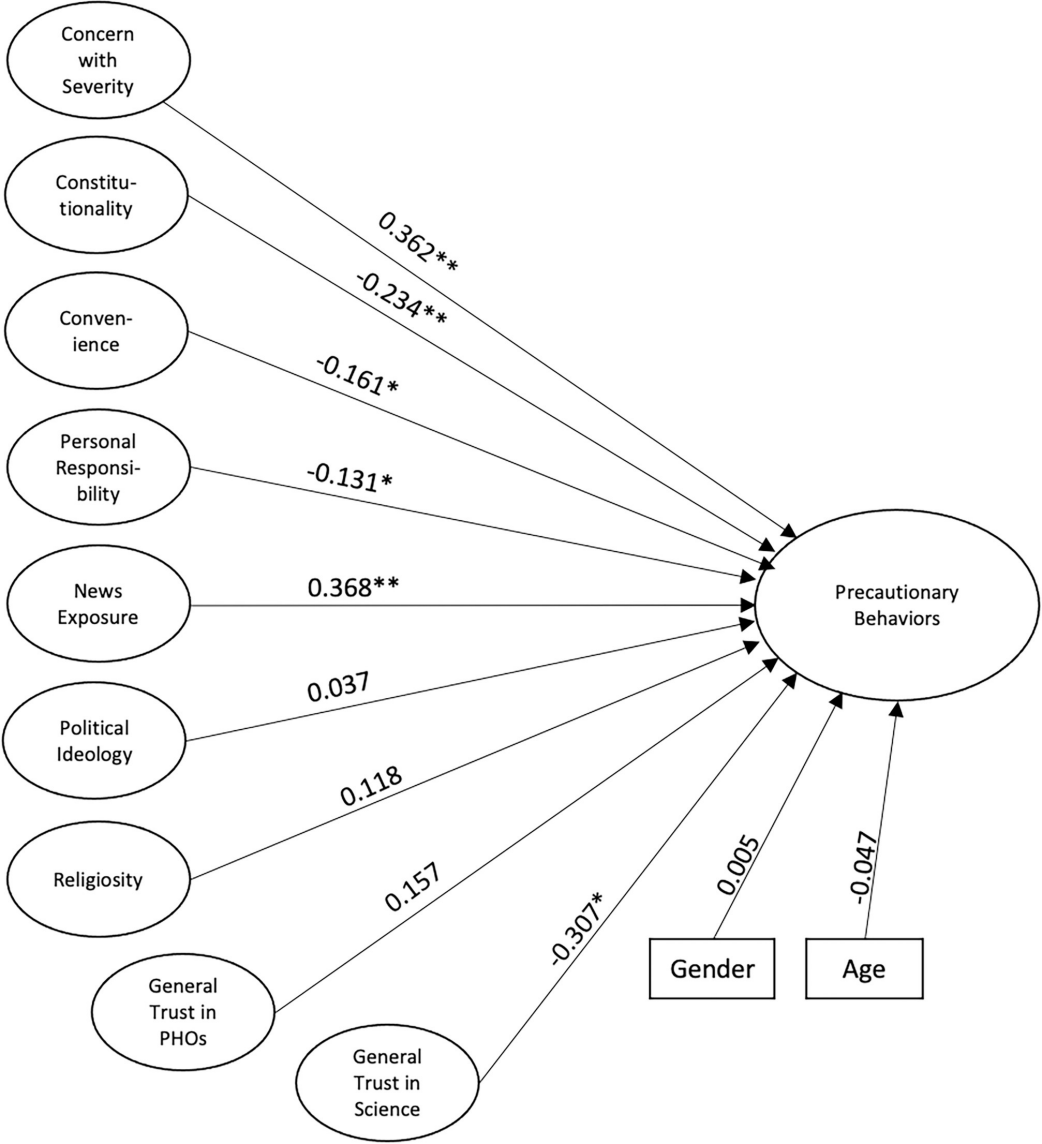
Structural equation model for the general population. The structural equation model for the General Population characterizes relationships between factors. Lines indicate predictive relationships. Significance is indicated *p < .05, **p < .001.

Fitting our hypothesized model resulted in unacceptable fit statistics for the General population (RMSEA = 0.049; CFI = 0.867; TLI = 0.867; SRMR = 0.166). However, removing the mediation of political ideology and religiosity through trust in science and public health officials resulted in robust fit to the data (RMSEA = 0.036; CFI = 0.936; TLI = 0.931; SRMR = 0.053). Model results indicated that concern about the severity of COVID-19, and news exposure both positively predicted precautionary behaviors. In contrast, feelings of unconstitutionality, impressions of convenience, feelings that it should be everyone’s personal responsibility to keep themselves safe, and general trust in science negatively correlate with students’ willingness to practice precautionary behaviors. Surprisingly, political ideology did not affect behaviors. We included both gender and age in the model as covariates, but neither were significant. The structural model is shown in Fig 4.

**Fig 5:**
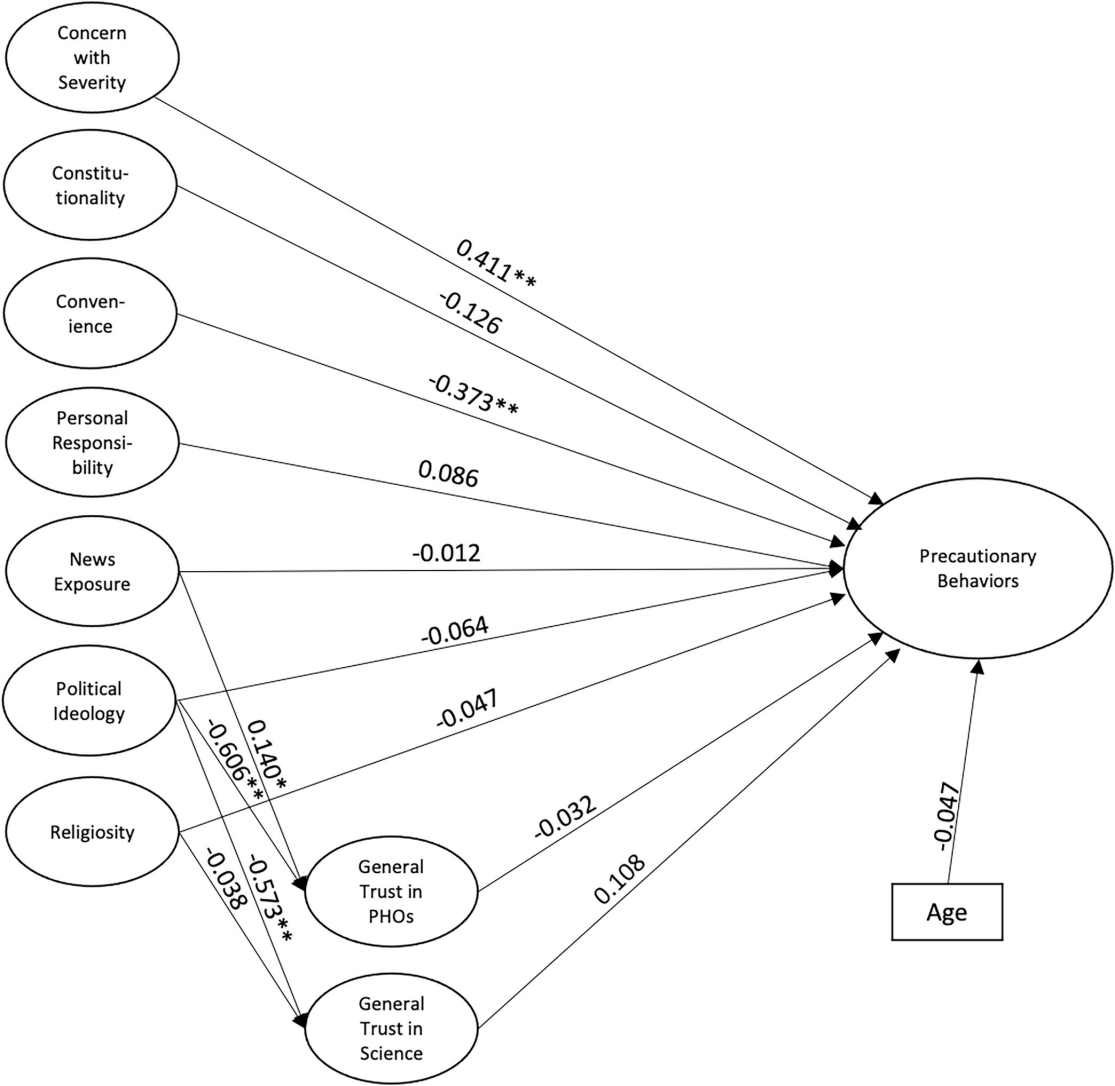
Structural equation model for the Utah population. The structural equation model for the Utah Population characterizes relationships between factors. Lines indicate predictive relationships. Significance is indicated *p < .05, **p < .001.

The hypothesized model (with mediation factors) fit the data for the Utah County population (RMSEA = 0.031; CFI = 0.952; TLI = 0.948; SRMR = 0.044). The only positive predictor of taking precautionary behaviors was a concern with severity. The only negative predictor was feelings of inconvenience. In other words, those who felt that the disease impact is more severe were more likely to take precautionary behaviors, whereas those not wearing a mask are most likely refraining out of inconvenience. Additionally, although trust in science and trust in public health officials did not have an impact on precautionary behaviors, both news exposure and political ideology positively correlated with students’ trust in public health officials (individuals reporting less news exposure and more conservative individuals had less trust in public health officials) and political ideology correlated with students’ trust in science (more conservative individuals were less likely to trust science). We included age in the model as a covariate but it was not significant (gender was not asked on the Utah County population survey). Again, the structural model is shown in Fig 5.

### Comparisons between populations

**Fig 6:**
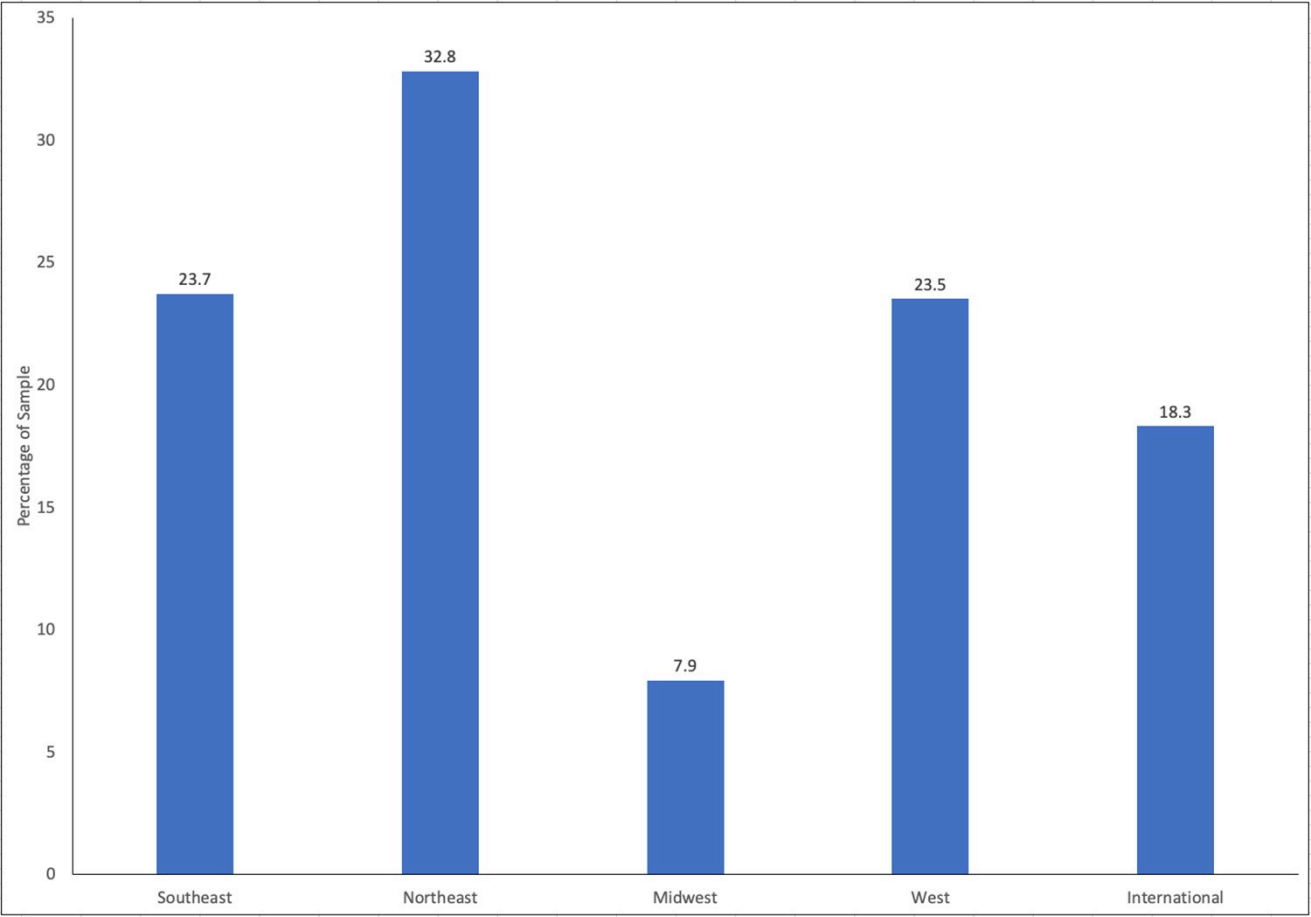
Summary of population distribution of the national sample. Regions are defined by: https://www.nationalgeographic.org/maps/united-states-regions/.

The Utah County sample was earlier in their college careers (p < .001), and younger (p < .001) than the general population (Table 6). The distribution of the national sample is shown in Fig 6.

**Table 6:** Comparison of population demographics.

Predictor	Scale	Mean	SD	p
Year (general)	N/A	3.31	1.41	< .001*
Year (Utah)		1.71	1.08	
Age (general)	N/A	27.61	6.22	< .001*
Age (Utah)		20.29	3.21	
Percent Female (general		.40		

This table reports the age, year in college and portion of the general population that is female in the general population. p-values of Wilcoxon Signed-Rank Tests comparing age and year in college are given.

*Denotes statistical significance.

## Discussion

We ran SEM analyses separately on the two student populations surveyed: students within Utah County and students across the nation. Although much of our discussion is focused on the differences between populations, we feel the uniqueness of the Utah County population is important. Our data demonstrates that nationwide opinions on COVID-19 data may not reflect the opinions and subsequent compliance with NPIs of students in various localities. Nonetheless, we feel the national data is a valuable resource for anyone trying to understand shifting opinions on COVID-19.

### Significant predictors

Within the nationwide survey of college students, two of the strongest predictors for compliance with NPIs were a concern with the severity of the COVID-19 pandemic and exposure to news (Fig 4). This suggests that a potential method for achieving widespread compliance with precautionary behaviors could be an increase in media promoting the behaviors and warning of the severity of the pandemic. Another significant predictor in the general population was concern that mask mandates violate constitutional rights. Those individuals with stronger beliefs that their personal constitutional rights and liberties were being infringed upon by COVID regulations were less likely to comply with NPIs. The constitutionality of mask mandates has been largely debated in the news; thus, this result is not surprising [57]. It may also be due to some of the more nuanced political views of young conservatives For example, a recent Pew poll found that Gen Z Republicans are more likely to say that blacks are treated less fairly than whites, the government should take action to control climate change, climate change is caused by anthropogenic activity, the government should solve problems, and that President Trump performed poorly compared to older Republicans [58].

Also significant in predicting compliance were feelings of convenience, opinions toward personal responsibility, and trust in science. Regarding convenience, the way in which our questions were worded on the survey may lead to the inevitable conclusion that those who find mask wearing inconvenient would likely not comply. This is still a concern that could be addressed by efforts to make compliance more convenient for students. Interestingly, those who feel that one’s health and safety is one’s own personal responsibility were less likely to practice NPIs. Thus, some of the pushback to mask wearing or social distancing may be a misunderstanding of how much one’s own actions may infringe upon others. In the Utah population, results differed slightly (Fig 5). There was a strong positive correlation between concern with severity and precautionary behaviors, but a strong negative correlation between convenience and precautionary behaviors. This suggests that in the Utah population, if compliance with mask wearing is deemed inconvenient, then individuals likely will not comply. Interestingly, the other correlations within the general population were not present in the Utah County population.

We also found a direct relationship between exposure to news and political ideology and one’s trust in public health officials, and between political ideology and trust in science. The correlation with news exposure is surprising and may suggest that the news media is portraying public health officials in a negative light, given that overall trust in public health officials has remained relatively high and even increased during the COVID pandemic [59]. It could also be due to the many changes in public health policies as the pandemic progressed and as more knowledge was gained that may lead people to believe the public health information is unreliable [60, 61]. The ties between political ideology and trust in public health officials and science is not surprising. The relationship between conservatism and a distrust of science has been well-documented [62, 63].

### Non-significant predictors

Aspects of our research were surprising and some even contrary to our initial predictions. For example, political ideology was not a predictor of compliance in either the Utah population or the general population. Although contrary to polling from earlier in the pandemic, this is in line with another recent publication which also found no strong correlation between political ideology and attitudes towards a future COVID-19 vaccine [64]. Age was also not a predictor of compliance with precautionary behaviors in either population; however, age was rather homogenous between populations due to our targeted sample. Gender was not a predictor in the general population (gender was not measured in the Utah population).

We were especially surprised that the general trust in science was not a strong predictor of compliance in the Utah County population, although it was somewhat negative in the general population. Based on rationale provided by Nadelson et al. [51], we believed that mistrust in science (and medical science in particular) stemmed from a lack of understanding of how the scientific process operates. The lack of strong correlation in either population may be due to the demographic we were surveying. We propose that perhaps because our population of college students likely had a similar basic level of exposure to scientific learning, we did not have enough variation in our data to detect meaningful correlations between trust in science and compliance to NPIs. Religiosity was not correlated with compliance with precautionary behaviors in the either population. The religious demographic in Utah County is predominantly members of the Church of Jesus Christ of Latter-Day Saints (CJCLDS) [65]. It comes as no surprise that a more nuanced range of beliefs within a relatively homogenous religious culture were displayed. The Utah population differed from the general population in that the factors constitutionality and exposure to news were not significantly correlated with compliance.

### Unique characteristics of populations

With roughly half our survey given to students living in Utah County and the other half given to students spread out across the nation, we expected the populations to differ moderately. As we take a deeper look into the differences between the two populations, we feel it is important to re-emphasize that universities across the country should consider the implications this could have to their own hometown, university, or area of residence. As different as the Utah findings are and the way in which the instrument functioned differently from the rest of the United States, we suspect a multitude of uniquely different pockets of opinions nationwide.

The demographics of our sample from Utah County were markedly different than those of the general population (Table 6), which could explain some of the differences seen in correlates (Figs 4 and 5). We believe the difference in these factors may stem from the large age difference between populations. The mean age in our Utah sample was 20.3, compared to 27.6 from the general sample (*p <* .001).

The cultural climate in Utah may explain the difference in the way the instrument functioned between populations. As has been mentioned earlier, many of those surveyed in Utah were members of the CJCLDS [66]. Like typical university students, those we surveyed in Utah live with roommates and potentially have family members who live close by. Unlike a typical university, students in Utah are likely part of a highly active church community, as well. A 2016 Pew research poll found that Latter-Day Saints (CJCLDS) were among the most highly involved with their congregations [67], and church members have been described by experts as “the most pro-social members in American society” [68]. It is possible that due to the sociality of this culture, a “small group” or “bubble” could be interpreted differently than the rest of America. It is important to note that officials of the Church (CJCLDS) in Utah have encouraged members to follow guidelines [69, 70].

During July of 2020 CJCLDS members residing in Utah valley received a letter from their regional church leaders strongly encouraging “all Latter-Day saints in the Utah Area to be good citizens by wearing face coverings while in public” [70]. This advice early on in the pandemic likely correlates to some of the trends we see now. hile we can only speculate at this time, the encouragement by church leaders provides a compelling reason for many Latter-Day Saints to comply publicly with NPIs.

Another interesting finding was that exposure to news was a significant predictor of compliance in the general population but not in the Utah population. It is possible that the Utah population watched and read different news sources. A recent poll found that 56% of Utahns get their news online [71] compared to 37% of all Americans [72]. Research has demonstrated that news sources can have a large influence on taking precautionary behaviors [73].

At the time the survey was administered political tension was high. Some news outlets around the time of the election reported skepticism of Latter-day Saints towards President Donald Trump [74, 75]. We submit this as one possible explanation for the less than expected conservatism among the traditionally more conservative Utah population. The students surveyed in Utah were selected from a mix of non-major and major specific introductory biology classes. Consequently, we suggest that this population may have had a high proportion of science majors. While we are unaware of any studies looking at undergraduate biology students in particular, it is noteworthy that the scientific community is more left-leaning than the public as a whole [76], and was much more likely to support then-Democratic presidential candidate Joe Biden [77]. In addition several major journals in the field of biology endorsed then Democratic candidate Joe Biden for president of the United States [78, 79]. It is possible that these factors could have tilted our Utah sample to the left, ideologically. The last explanation for the more liberal Utah population is the age difference. A 2019 Pew research poll found Generation Z to lean a little more liberal than older generations, a difference especially pronounced among Gen-Zers who identify as Republican [58].

### Limitations

A quick search of a popular government funded medical search engine reveals over 75,000 published papers on COVID-19 thus far. Due to the rapidly evolving nature of the pandemic, we recognize that any survey published on the subject will be highly specific to the time frame it was published in. The survey was administered from October 14th to November 10^th^, 2020, in the midst of a heated United States presidential election. According to recent research, risk perception of COVID-19 is based highly upon the statements and actions of political leaders [54]. During the time of an election, individuals are often highly involved in politics, thus their views on COVID-19 may be influenced by politics more than usual. This was before Pfizer [80] and Moderna [81] had released promising phase 3 results about their vaccines. We acknowledge that these events may have, as of yet, not understood consequences into the modeling of our compliance with NPIs.

Additionally, we recognize some limitations to our sampling. Only 40 percent of our national population was female, and we did not collect information on gender amongst our Utah Valley population. Thus, it is highly likely that the gender composition between these populations differed. However, it is important to note that gender was a non-significant predictor in our general population. Our student samples were drawn entirely from freshman-level biology courses at two universities. These populations were the most convenient for us to sample on a consistent level. The courses are generally required for all students, but many of the students are on a life sciences track. It’s possible the samples we pulled are not perfectly representative of the student population as a whole.

Lastly, it is important to note that this study is correlational. Thus, any conclusions drawn regarding relationships should be taken with this precaution in mind. It is possible for potential causality between components in the model; however, these relationships warrant further experimentation.

## Conclusions

The results from our survey of college students across the country can serve as a framework for universities to increase compliance. While we have not conducted any research on interventions, it is reasonable to conclude that addressing these predictors would increase the efficacy of interventions. It is important to note that predictors in our local sample significantly differed from our national population in the number of predictive factors affecting compliance. This suggests that it is highly likely that interventions at individual universities will be most effective when based upon data gathered in individual communities. For example, if officials in Utah were to base interventions on our national data, they may overemphasize sharing generic COVID news, and underemphasize increasing the convenience of compliance, making their interventions less effective than they could be.

Vaccines are now available to the general public; however, vaccination compliance is still a challenge [82]. We call on universities across the country to survey their students, and better understand why they may, or may not, be compliant with NPIs. If this is not possible, we suggest they apply our general results, and results from other nationwide surveys. We believe doing so could be an important step for mitigating spread on campuses, and the possible overflow into surrounding populations.

Unfortunately, this will likely not be the last public health emergency or pandemic in the near future, especially as humans encroach further upon nature [83–86]. We believe it is vital to learn all we can about combatting this pandemic, and we call on researchers across the globe to work on understanding predictors of compliance, so interventions can be prepared for the next outbreak.

## Supporting information

S1 File (DOCX)
