# Supplementary material for: Predictors of compliance with COVID-19 related non-pharmaceutical interventions among university students in the United States
Source: PLoS One. 2021 Jun 18;16(6):e0252185. doi: 10.1371/journal.pone.0252185 (PMC8213108; doi:10.1371/journal.pone.0252185)
Supplement: S1 File — (DOCX) [file pone.0252185.s001.docx]

COVID Survey - Precautionary Behaviors - General

Start of Block: Demographic questions

Are you currently enrolled in college?

- Yes
- No

Skip To: End of Block If Are you currently enrolled in college? = No

What year in college is this for you?

- 1
- 2
- 3
- 4
- 5
- 6+

| 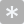 |
| --- |

What is your age?

________________________________________________________________

| Page Break |  |
| --- | --- |

How do you identify?

- Male
- Female
- Other

What race/ethnicity do you identify as?

- White
- African American
- Hispanic
- Asian
- American Indian or Alaska Native
- Native Hawaiian or Pacific Islander
- Other

Where are you from?

▼ Alabama ... Outside the US

| Page Break |  |
| --- | --- |

What type of institution do you attend?

- Private 2 year college
- Private 4 year college
- Private trade school
- Public 2 year college
- Public 4 year college
- Public trade school

Where is your institution located

▼ Alabama ... Outside the US

Which of the following best describes your current situation?

- All of my classes are in-person on campus
- At least part of my classes are in-person, and the others are online
- All of my classes are online but I still take part in campus activities
- All of my classes are online and I am remote, so I don't participate in any campus activities

End of Block: Demographic questions

Start of Block: Political ideology

What political party do you most identify with?

- The Democratic Party
- The Republican Party
- Neither

On a scale of 1 to 7, 1 being very left (or liberal) leaning and 7 being very right (or conservative) leaning, how would you rate your views of each category of issues?

|  | 0 | 1 | 2 | 3 | 4 | 5 | 6 | 7 |
| --- | --- | --- | --- | --- | --- | --- | --- | --- |

| Economic Issues | 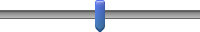 |
| --- | --- |
| Social Issues | 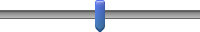 |
| Scientific Issues | 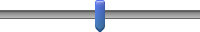 |

End of Block: Political ideology

Start of Block: Religiosity

Before the emergence of COVID-19, how often did you attend church or other religious meetings?

- Never
- Once a year or less
- A few times per year
- A few times per month
- Once a week
- More than once a week

How often do you spend time in private religious activities, such as prayer, meditation, or scripture study?

- Rarely or Never
- A few times per month
- Once per week
- Two or more times per week
- Daily
- More than once per day

In my life, I experience the presence of the Divine (i.e., God)

- Definitely not true
- Tends not to be true
- Unsure
- Tends to be true
- Definitely true of me

My religious beliefs are what really lie behind my whole approach to life. 

- Definitely not true
- Tends not to be true
- Unsure
- Tends to be true
- Definitely true of me

I try hard to carry my religion over into all other dealings in life

- Definitely not true
- Tends not to be true
- Unsure
- Tends to be true
- Definitely true of me

End of Block: Religiosity

Start of Block: Religious Beliefs and COVID-19

My religion influences my beliefs beliefs about the current covid-19 pandemic

- Definitely not true
- Tends not to be true
- Unsure
- Tends to be true
- Definitely true of me

My religion influences my approach to the current covid-19 pandemic

- Definitely not true
- Tends not to be true
- Unsure
- Tends to be true
- Definitely true of me

My religious beliefs shape how I have interacted with others during the COVID-19 pandemic

- Definitely not true
- Tends not to be true
- Unsure
- Tends to be true
- Definitely true of me

End of Block: Religious Beliefs and COVID-19

Start of Block: Constitutionalism

How much do you agree with the following statements:


I don’t mind masks, but mask mandates are unconstitutional.

- Strongly agree
- Somewhat agree
- Neither agree nor disagree
- Somewhat disagree
- Strongly disagree

Mask mandates interfere with my constitutional rights.

- Strongly agree
- Somewhat agree
- Neither agree nor disagree
- Somewhat disagree
- Strongly disagree

Mask mandates interfere with my personal freedoms.

- Strongly agree
- Somewhat agree
- Neither agree nor disagree
- Somewhat disagree
- Strongly disagree

| Page Break |  |
| --- | --- |

COVID-19 restrictions restrict my constitutional rights.

- Strongly agree
- Somewhat agree
- Neither agree nor disagree
- Somewhat disagree
- Strongly disagree

I have the constitutional right to refuse to wear a mask in public.

- Strongly agree
- Somewhat agree
- Neither agree nor disagree
- Somewhat disagree
- Strongly disagree

Wearing a mask should be a personal choice, not a legal mandate.

- Strongly agree
- Somewhat agree
- Neither agree nor disagree
- Somewhat disagree
- Strongly disagree

| Page Break |  |
| --- | --- |

Please rank the following priorities of public health officials in order of importance, from most important to least important

______ Treat everyone as equally as possible

______ Protect the health of the greatest number of people

______ Give priority to sick and frail people in getting attendance

______ Aim to preserve essential community services like electricity and law enforcement

______ Do not interfere with the civil liberties or freedoms of people in your communities

This is a control question, mark agree and move one

- Agree
- Disagree

End of Block: Constitutionalism

Start of Block: Convenience

How true are the following statements for you?


The discomfort of a mask is enough to make me not wear them.

- Definitely not true
- Tends not to be true
- Unsure
- Tends to be true
- Definitely true of me

Physical issues (e.g., glasses fogging up, facial breakouts, difficulty breathing) prevent me from wearing a mask.

- Definitely not true
- Tends not to be true
- Unsure
- Tends to be true
- Definitely true of me

The lack of availability of masks prevents me from wearing one.

- Definitely not true
- Tends not to be true
- Unsure
- Tends to be true
- Definitely true of me

Forgetting to bring a mask with me is often a reason I don’t wear one.

- Definitely not true
- Tends not to be true
- Unsure
- Tends to be true
- Definitely true of me

End of Block: Convenience

Start of Block: Exposure to News

How often do you check COVID-19 related news?

- Everyday
- A few times a week
- A few times a month
- About once a month
- Every few months or less
- Never

How often do you check scientific news in general?

- Everyday
- A few times a week
- A few times a month
- About once a month
- Every few months or less
- Never

How often do you check non science-specific news in general?

- Everyday
- A few times a week
- A few times a month
- About once a month
- Every few months or less
- Never

End of Block: Exposure to News

Start of Block: Precautionary Behaviors

How often do you wear a mask in places where you are required to (i.e. grocery stores, schools)?

- Always
- Almost always
- Sometimes
- Rarely
- Never

How often do you wear a mask when you are with a small group (10 people or less) of friends, family, etc. (individuals you don’t live with but see often)?

- Always
- Almost always
- Sometimes
- Rarely
- Never

How often do you wear a mask when you are in a public outdoor space within six feet of others?

- Always
- Almost always
- Sometimes
- Rarely
- Never

| Page Break |  |
| --- | --- |

How often do you gather in a group of more than 10 people and not wear a mask?

- Always
- Almost always
- Sometimes
- Rarely
- Never

Outside of your private living space, how often do you practice social distancing (staying 6 feet or more from others)?

- Always
- Almost always
- Sometimes
- Rarely
- Never

| Page Break |  |
| --- | --- |

Have your handwashing habits changed since the start of the COVID-19 pandemic?

- I have been washing my hands a lot more
- I have been washing my hands somewhat more
- I haven't changed my handwashing habits
- I have been washing my hands somewhat less
- I have been washing my hands a lot less

Has the frequency with which you sanitize your spaces (desks, chairs, phone, computer) changed since the start of the COVID-19 pandemic?

- I have been sanitizing my surfaces a lot more
- I have been sanitizing my surfaces somewhat more
- I haven't changed the frequency with which I sanitize personal spaces
- I have been sanitizing my surfaces somewhat less
- I have been sanitizing my surfaces a lot less

End of Block: Precautionary Behaviors

Start of Block: Trust in Science and Scientists

To what degree do you agree with the following statements?


Scientists ignore evidence that contradicts their work.

- Strongly Agree
- Agree
- Neither agree nor disagree
- Disagree
- Strongly Disagree

Scientific theories are weak explanations.

- Strongly Agree
- Agree
- Neither agree nor disagree
- Disagree
- Strongly Disagree

Scientists intentionally keep their work secret.

- Strongly Agree
- Agree
- Neither agree nor disagree
- Disagree
- Strongly Disagree

| Page Break |  |
| --- | --- |

Scientists don’t value the ideas of others.

- Strongly Agree
- Agree
- Neither agree nor disagree
- Disagree
- Strongly Disagree

We should trust the work of scientists.

- Strongly Agree
- Agree
- Neither agree nor disagree
- Disagree
- Strongly Disagree

We should trust that scientists are being honest in their work.

- Strongly Agree
- Agree
- Neither agree nor disagree
- Disagree
- Strongly Disagree

| Page Break |  |
| --- | --- |

We should trust that scientists are being ethical in their work.

- Strongly Agree
- Agree
- Neither agree nor disagree
- Disagree
- Strongly Disagree

Scientific theories are trustworthy.

- Strongly Agree
- Agree
- Neither agree nor disagree
- Disagree
- Strongly Disagree

We can trust science to find the answers that explain the natural world.

- Strongly Agree
- Agree
- Neither agree nor disagree
- Disagree
- Strongly Disagree

| Page Break |  |
| --- | --- |

We cannot trust scientists because they are biased in their perspectives.

- Strongly Agree
- Agree
- Neither agree nor disagree
- Disagree
- Strongly Disagree

Scientists will protect each other even when they are wrong.

- Strongly Agree
- Agree
- Neither agree nor disagree
- Disagree
- Strongly Disagree

We cannot trust scientists to consider ideas that contradict their own.

- Strongly Agree
- Agree
- Neither agree nor disagree
- Disagree
- Strongly Disagree

| Page Break |  |
| --- | --- |

Today’s scientists will sacrifice the well being of others to advance their research.

- Strongly Agree
- Agree
- Neither agree nor disagree
- Disagree
- Strongly Disagree

We cannot trust science because it moves too slowly.

- Strongly Agree
- Agree
- Neither agree nor disagree
- Disagree
- Strongly Disagree

This is a control question, please selection option A and move on

- A
- B
- C
- D

End of Block: Trust in Science and Scientists

Start of Block: Trust in Public Health Authorities

For this next section, you will see the term ‘public health authority’ used a number of times. We are using this term to mean individuals employed by tax-funded government health agencies, whether that be local, state, or federal. Specifically, a public health authority communicates with the public with statements about health concerns and recommended health practices. A prominent example would be Anthony Fauci, M.D., director of NIAID (National Institute of Allergy and Infectious Diseases). 

How much do you agree with the following statements?


Public health authorities intentionally keep some information secret.

- Strongly Agree
- Agree
- Neither agree nor disagree
- Disagree
- Strongly Disagree

Public health authorities are seeking to fulfill an agenda.

- Strongly Agree
- Agree
- Neither agree nor disagree
- Disagree
- Strongly Disagree

Public health authorities don’t value the ideas of others.

- Strongly Agree
- Agree
- Neither agree nor disagree
- Disagree
- Strongly Disagree

| Page Break |  |
| --- | --- |

I trust that public health authorities want to make life better for people.

- Strongly Agree
- Agree
- Neither agree nor disagree
- Disagree
- Strongly Disagree

We should trust that public health authorities are being honest in their work.

- Strongly Agree
- Agree
- Neither agree nor disagree
- Disagree
- Strongly Disagree

We should trust that public health authorities are being ethical in their work.

- Strongly Agree
- Agree
- Neither agree nor disagree
- Disagree
- Strongly Disagree

| Page Break |  |
| --- | --- |

When public health authorities give health instruction, they are just guessing.

- Strongly Agree
- Agree
- Neither agree nor disagree
- Disagree
- Strongly Disagree

We cannot trust public health authorities because they are biased in their perspectives.

- Strongly Agree
- Agree
- Neither agree nor disagree
- Disagree
- Strongly Disagree

We cannot trust public health authorities to consider ideas that contradict their own.

- Strongly Agree
- Agree
- Neither agree nor disagree
- Disagree
- Strongly Disagree

| Page Break |  |
| --- | --- |

Today’s public health authorities will sacrifice the well being of others to advance their agenda.

- Strongly Agree
- Agree
- Neither agree nor disagree
- Disagree
- Strongly Disagree

When public health authorities provide conflicting advice, it diminishes my trust in their work.

- Strongly Agree
- Agree
- Neither agree nor disagree
- Disagree
- Strongly Disagree

Public health authorities ignore evidence that contradicts their work.

- Strongly Agree
- Agree
- Neither agree nor disagree
- Disagree
- Strongly Disagree

End of Block: Trust in Public Health Authorities

Start of Block: Impression of Severity

How concerned are you with getting COVID-19?

- Extremely concerned
- Very concerned
- Moderately concerend
- Somewhat concered
- Not at all concerend

How do you think getting COVID-19 would affect you physically?

- It would make me extremely sick
- It would make me very sick
- It would make me moderately sick
- It would make me somewhat sick
- It would not make me sick at all

How do you think others in your community would be effected by a positive COVID-19 case?

- Many would get very sick leading to hospitalizations and death.
- A fair amount of people would get sick, leading to some hospitalizations and death.
- Some individuals would get sick, leading to a small number of hospitalizations and deaths.
- Most people would be fine, only a relatively few would get sick, and these are those who would have gotten sick otherwise.
- There would be no spread of sickness and there would not be an increase of hospitalizations and death.

How do you think getting COVID-19 would affect other aspects of your life?

- It would heavily affect all aspects of my life.
- It would affect all aspects of my life in some way.
- It would affect many aspects of my life.
- It would be an inconvenience but I would mostly live normally.
- It would not affect my life at all.

This is a control question, please select option D and move on

- A
- B
- C
- D

End of Block: Impression of Severity

Start of Block: Personal Responsibility

On a scale of 1 to 7, 1 being strongly agree and 7 being strongly disagree, rank the following statements on how much you agree with them:

|  | Strongly Agree | Agree | Somewhat agree | Neither agree nor disagree | Somewhat disagree | Disagree | Strongly disagree |
| --- | --- | --- | --- | --- | --- | --- | --- |

|  | 0 | 1 | 2 | 3 | 4 | 5 | 6 | 7 |
| --- | --- | --- | --- | --- | --- | --- | --- | --- |

| “I shouldn’t be mandated to wear a mask because those who are at high risk for contracting the virus should be responsible for keeping themselves safe.” | 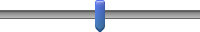 |
| --- | --- |
| "I am responsible for the health of others in my community." | 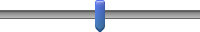 |
| "Everyone should take responsibility for their own health and act accordingly." | 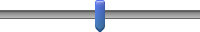 |
| "Those who are at high risk should stay out of public places, rather than having everyone else take precautions for their health." | 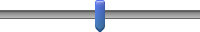 |
| A | 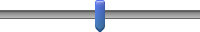 |

End of Block: Personal Responsibility
